# Identification of tell-tale patterns in the 3′ non-coding region of hantaviruses that distinguish HCPS-causing hantaviruses from HFRS-causing hantaviruses

**DOI:** 10.1038/s41426-018-0027-z

**Published:** 2018-03-21

**Authors:** Sathish Sankar, Jayanta Borkakoti, Mageshbabu Ramamurthy, Balaji Nandagopal, Perumal Vivekanandan, Sridharan Gopalan

**Affiliations:** 1 Sri Sakthi Amma Institute of Biomedical Research, Sri Narayani Hospital and Research Centre, Sripuram, Vellore, 632055 Tamil Nadu India; 2 Kusuma School of Biological Sciences, Indian Institute of Technology, New Delhi, 110016 India

Hantaviruses are negative-sense, single-stranded RNA viruses with small (S)-, middle (M)-, and large (L)-segments. Each segment has a coding region and non-coding regions (NCRs)^1^. Hantaviruses associated with human diseases specifically cause either hemorrhagic fever with renal syndrome (HFRS) or hantavirus cardiopulmonary syndrome (HCPS)^2^. A few hantaviruses have been associated with both syndromes. Efforts to identify differences in amino-acid sequences and nucleotide sequences between hantaviruses causing HFRS and HCPS have failed. Clinically, the need to distinguish hantaviruses based on their disease-causing ability is being increasingly recognized^3^ because of the discovery of several new hantaviruses in the last decade. In addition, differences between hantavirus genotypes in susceptibility to antiviral agents are well-documented. For example, ribavirin is useful for treating HFRS, but not HCPS^4^. Favipiravir may be useful for treating HCPS^5^. However, specific genomic differences, if any, between HFRS-causing hantaviruses and HCPS-causing hantaviruses have yet to be elucidated.

Previous studies on other RNA viruses indicate important roles for the 3′ NCRs in virus replication and infectivity^6^. Among hantaviruses, the nucleotide sequence of the 3′ NCR of the S-segment is highly variable^1^, making meaningful sequence comparisons of the 3′ NCR extremely challenging^7^. Therefore, there is a paucity of reports that systematically analyze the 3′ NCR of hantaviruses. In this study, we investigated the association, if any, between the length, CpG content, and RNA-folding free energy of hantavirus NCRs and the ability of hantaviruses to cause HFRS, HCPS, or both. Our results highlight an important and yet unknown link between the length and CpG content of hantavirus 3′ NCR and human disease.

The S-, M-, and L-segments of hantavirus sequences with complete coding DNA sequences along with the 3′ NCR and 5′ NCR were obtained from GenBank. The accession numbers of all 896 sequences used for analysis are given in Supplementary Table S1. Viruses reported in the International Committee on Taxonomy of Viruses classification and fulfilling the above criteria were selected and used for further analysis. The reservoir hosts for Old World hantaviruses are primarily restricted to Eurasia, while the reservoirs for New World hantaviruses are found in the Americas^2^. The sequences analyzed were classified into four categories (HCPS only, HFRS only, both HCPS and HRFS, and unknown) based on their ability to cause human disease (Supplementary Fig. S2).

We analyzed the length of the 3′ NCR (*n* = 896) and 5′ NCR (*n* = 631) of hantavirus sequences using BioEdit v7.2.5 (http://www.mbio.ncsu.edu/bioedit/page2.html). Data were analyzed using Student’s *t*-test. Box plots were made using MS-Excel. The results were considered statistically significant at a *P*-value of <0.05.

The lengths of the 3′ NCRs of the S- and L-segments were significantly longer in New World hantaviruses than Old World hantaviruses (Fig. 1a, b; *P < *0.0001). The existence of such striking differences in the 3′ NCR lengths between New world hantaviruses and Old World hantaviruses has not been reported previously. The length of the 3′ NCR of the M-segment was comparable between New World hantaviruses and Old World hantaviruses (Supplementary Fig. S1A).

**Fig. 1:**
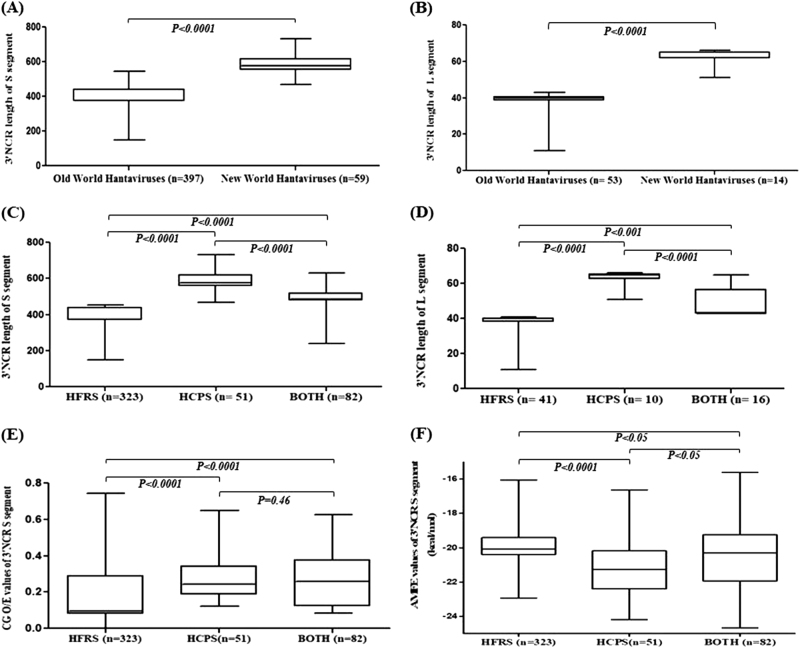
**Differences in the 3´ NCR lenghts, CpG content and adjusted minimum free energy (AMFE) values among hantaviruses**. Box plots showing the distribution of 3′ NCR length among the Old World Hantaviruses and New World Hantaviruses in" and please revise this figure legend as follows:Box plots showing the distribution of 3′ NCR length among the Old World Hantaviruses and New World Hantaviruses in **(a)** the S- segment and **(b)** the L- segment. Box plots showing the distribution of 3′ NCR length of **(c)** the S- segment and **(d)** the L- segment among hantaviruses causing HCPS, HFRS, or both. Box plots showing the distribution of **(e)**CpG_O/E_ values and **(f)** adjusted minimum free energy (AMFE) values for the 3′ NCR of the S- segment. "?>Box plots showing the distribution of 3′ NCR length among the Old World Hantaviruses and New World Hantaviruses in a the S-segment and **b** the L-segment. Box plots showing the distribution of 3′ NCR length of **c** the S-segment and **d** the L-segment among hantaviruses causing HCPS, HFRS, or both. Box plots showing the distribution of **e** CpG_O/E_ values and **f** adjusted minimum free energy (AMFE) values for the 3′ NCR of the S-segment

We then investigated, an association, if any, between the length of the 3′ NCR of the S-, M-, and L-segments and the ability of hantaviruses to cause HCPS, HFRS, or both. Interestingly, the 3′ NCRs of the S- and L-segments for HCPS-causing hantavirus genotypes were significantly longer than that for HFRS-causing hantavirus genotypes (Fig. 1c, d; *P < *0.0001 and *P < *0.0001, respectively; Supplementary Table S3). The length of the 3′ NCR of the M-segment was comparable between HFRS-causing and HCPS-causing genotypes (Supplementary Fig. S1B; *P* = 0.77). The number of sequences available for analysis varied greatly across the hantaviruses analyzed. The results from analysis of a maximum of five sequences each for a given hantavirus (Supplementary Fig. S2) are in keeping with that from analyzing all available sequences (Fig. 1c, d). The variability of the 3′ NCR sequence lengths in hantaviruses is well-documented for the S-segment^7^; nonetheless, the potential of the 3′ NCR length to distinguish HCPS-causing genotypes from HFRS-genotypes was not known previously.

The average length of the 5′ NCR sequences analyzed are tabulated in Supplementary Table S4. The 5′ NCRs of the hantavirus M-segment of HCPS-causing genotypes were significantly longer than those of the HFRS-causing genotypes (Supplementary Table S4).

Our findings clearly demonstrate that the 3′ NCRs of the S- and the L-segments are longer in HCPS-causing genotypes than in HFRS-causing genotypes. HFRS is caused by Old World hantaviruses, and HCPS is caused by New World hantaviruses^2^. It is possible that the differences in the NCR lengths of hantavirus genotypes causing HFRS and HCPS may reflect the differences between Old World and New World hantaviruses. The hantaviruses that cause both HFRS and HCPS include Old World viruses (Puumala virus and Tula virus) and New World viruses (Andes virus, Black creek Canal virus, and Bayou virus). If the observed differences in the lengths of the NCRs between hantaviruses are linked to the geographic distribution of the reservoir host (Old World vs. New World hantaviruses), one would expect that the NCR lengths of Puumala viruses and Tula viruses (both Old World hantaviruses), which can cause both HFRS and HCPS, would be comparable to those of other Old World viruses that cause HFRS. Interestingly, our results indicate that the 3′ NCRs of the S-segment of Puumala viruses and Tula viruses that cause both HFRS and HCPS are significantly longer than those of the rest of the Old World hantaviruses known to cause only HFRS (Supplementary Fig. S3A). In addition, the 3′ NCRs of the S-segment of hantaviruses causing HCPS only or both HCPS and HFRS were significantly longer than those causing HFRS only; this was independent of the geographic distribution of the Old World and New World hantaviruses.

Neither the length or the sequence of the 3′ NCR segment of a given hantavirus is amenable to major changes, suggesting a functional role for the 3′ NCR in hantavirus genomes^1^. Our results indicate major differences in the length of the 3′ NCR of the S-segment between hantaviruses causing HCPS and HFRS. We then analyzed (a) the relative abundance of CpG dinucleotides using methods described elsewhere^8^ and (b) the minimum free energy (MFE) for RNA folding, calculated using ViennaRNA Package 2.0 (http://www.tbi.univie.ac.at/RNA) for the 3′ NCR of the S-segment. Subsequently, the adjusted minimum free energy (AFME) was calculated using the formula AMFE = (100 x MFE)/(length of the RNA)^9^. The relative abundance of CpG dinucleotides should not be affected by the differences in the length of the 3′ NCR of the S-segment. AMFE is adjusted for length, so the differences in the length of the 3′ NCR of the S-segment between viruses would affect AMFE values to a lesser extent than MFE values. Both the CpG content^10,11^ and RNA-folding free energy^12^ in RNA viruses have been linked to virus pathogenesis. Interestingly, our data suggest the 3′ NCR of the S-segment of hantaviruses causing HCPS only or both HCPS and HFRS had higher CpG content (Fig. 1e) compared to those causing HFRS only; this was independent of the geographic distribution of the Old World and New World hantaviruses (Supplementary Fig. S3B). Although the differences in AMFE values between the 3′ NCR of the S-segment of hantaviruses causing HCPS only or both HCPS and HFRS were statistically significant (Fig. 1f and Supplementary Fig. S3C), the implications of such small differences on RNA stability is not fully understood.

The roles of the 3′ NCRs in virus pathogenesis and adaptation to hosts have been described for other RNA viruses^13,14^. Among hantaviruses, the NCR of the S-segment has the ability to initiate virus replication, that of the M-segment has the strongest promoter activity, and that of the L-segment has the best packaging efficiency^15^. We speculate that the observed differences in the length and RNA-folding free energy of the 3′ NCR sequences from the S-segment between HCPS-causing hantaviruses and HFRS-causing hantaviruses may influence the formation and the stability of RNA secondary structures. In addition, the observed differences in CpG content can potentially influence hantavirus adaptation to specific hosts or host cell types.

In sum, our findings have identified major differences in the 3′ NCR of the S-segment between hantaviruses causing HFRS and HCPS in (a) the length (b) the relative abundance of CpG dinucleotides, and (c) the RNA-folding free energy. None of these differences have been previously reported, and all of them have implications in virus pathogenesis. Taken together, our findings suggest that the observed differences in the 3′ NCR of the S-segment do not merely reflect the geographic distribution of the reservoir host but are linked to the specific syndromes caused by hantavirus genotypes in humans. The association between the tell-tale patterns of the 3′ NCR and specific clinical syndromes caused by hantaviruses merits further investigation. We believe that this work provides a novel perspective to our current understanding of the role of NCRs in the pathogenesis of hantaviruses.

## Electronic supplementary material

Supplementary Tables

Supplementary Figure S1

Supplementary Figure S2

Supplementary Figure S3
