# Supplementary material for: Identification of tell-tale patterns in the 3′ non-coding region of hantaviruses that distinguish HCPS-causing hantaviruses from HFRS-causing hantaviruses
Source: Emerg Microbes Infect. 2018 Mar 21;7:32. doi: 10.1038/s41426-018-0027-z (PMC5861111; doi:10.1038/s41426-018-0027-z)
Supplement: Supplementary file 1 — Supplementary Tables [file 41426_2018_27_MOESM1_ESM.doc]

**Supplementary Table S1** **Accession numbers of all the Hantaviruses sequences analysed.** Sequences obtained from hantaviruses infecting non-natural hosts (n=2) and sequences with non-coding regions of less than 5 nucleotides were excluded from analysis.

Virus names with the accession numbers analysed of the S segment

| Virus Names | Accession numbers |
| --- | --- |
| Hantaan virus | JQ083395.1,AF321095.1,AF321094.1,U37768.1,AY017064.1,AB027101.1,AB027097.1,AF288296.1,AF366568.1,GU329991.1,AF329390.1,  EU363813.1,EU363812.1,EU363811.1,EU363809.1,EU363808.1,  FJ753396.1,KC344236.1,EF990915.1,EF990914.1,EF990913.1,EF990912.1,EF990911.1,EF990910.1,EF990909.1,EF990908.1,AY839871.1,  AF427324.1,AF427323.1,AF427322.1,AF427320.1,AF427319.1,  AF427318.1,D25533.1,D25530.1,KM355414.1,KJ857348.1,KJ857347.1,  KC344249.1,KC344248.1,KC344247.1,KC344246.1,KC344245.1,  KC344244.1,KC344243.1,KC344242.1,KC344241.1,KC344240.1,  KC344239.1,KC344238.1,KC344237.1,JQ912770.1,JQ912769.1,  JQ912768.1,JQ912767.1,JQ912766.1,JQ912760.1,JQ912759.1,  JQ912758.1,JQ912757.1,JQ912756.1,JQ912755.1,JQ912754.1,  JQ912753.1,JQ912752.1,JQ912751.1,JQ912750.1,JQ912749.1,  JQ912748.1,JQ912747.1,JQ912746.1,JQ912745.1,JQ912744.1,  JQ912743.1,JQ912742.1,JQ912741.1,JQ912740.1,JQ912739.1,  JQ912738.1,JQ912737.1,JQ912736.1,JQ912735.1,JQ912734.1,  JQ912733.1,JQ912732.1,JQ912731.1,JQ912730.1,JQ912729.1,  JQ912728.1,JQ912727.1,JQ912726.1,JQ912725.1,JQ912724.1,  JQ912723.1,JQ912722.1,JQ912721.1,JQ912720.1,JQ912719.1,  JQ912718.1,JQ912717.1,JQ912716.1,JQ912715.1,JQ912714.1,  JQ912713.1,JQ912712.1,JQ912711.1,JQ912710.1,JQ912709.1,  JQ912708.1,JQ912707.1,JQ912706.1,JQ912705.1JQ912704.1,  JQ912703.1,JQ912702.1,JQ912701.1,JQ912700.1,JQ912699.1,  JQ912698.1,JQ912697.1,HQ611981.1,EF121324.1,KC570390.1,  KC570389.1,KC570388.1,KC570387.1,KC570386.1,KC570385.1,  KC570384.1,JQ665906.1,JQ665905.1,HQ834507.1,HQ834506.1,  HQ834505.1,HQ834504.1,HQ834503.1,HQ834502.1,HQ834501.1,  HQ834500.1,HQ834499.1,AB620031.1,AF288646.1,AF288644.1,  AB127998.1,DQ658415.1,EF595840.1,AY748309.1,AB027523.1,  NC_005218.1 |
| Dobrava-Belgrade virus | JF920150.1,AY168576.1,AY961615.1,AY961618.1,AY533120.2,  AY533118.2,NC_005233.1,AJ410615.1,AJ410619.1,EU188452.1,  EU188449.1,L41916.1,JQ026204.1,GQ205408.1,GQ205407.1,  GQ205406.1,GQ205405.1,GQ205404.1,GQ205403.1,GQ205402.1,  GQ205401.1,KC676608.1,KC676606.1,KC676603.1,KC676602.1,  KC676601.1,KC676600.1,KC676599.1,KC676598.1,KC676597.1,  KC676595.1,KC676592.1,KC676591.1,KC676590.1,KC676589.1,  GU904032.1,GU904031.1,GU904030.1,GU904029.1,GU904028.1,  GU904027.1 |
| Virus Names | Accession numbers |
| Seoul virus | AY006465.1,KC902522.1,KP645198.1,NC_005236.1,AY273791.1,  JX853575.1,AY750171.1,EF192308.1,AY766368.1,KF387725.1,  JX879769.1,AF406965.1,KM948598.1,GU361893.1,AB618112.1,  AF329389.1,AF329388.1,KJ950869.1,KJ950868.1KJ950867.1,  KJ950866.1,AY627049.2,KP859512.1,KP859511.1,JQ898106.1,  HQ611980.1,GU592953.1,GU592952.1,GU592951.1,GU592950.1,  GU592949.1,GU592948.1,GU592947.1,GU592946.1,GU592945.1,  GU592944.1,GU592943.1,GU592942.1GU592941.1,GU592940.1,  GU592939.1,GU592938.1,GU592937.1,GU592936.1,GU592935.1,  GU592934.1,GU592933.1,GU592932.1,GQ279395.1,GQ279394.1,  GQ279393.1,GQ279392.1,GQ279391.1,GQ279390.1,GQ279389.1,  GQ279388.1,GQ279387.1,GQ279386.1,GQ279385.1,GQ279384.1,  GQ279383.1,GQ279382.1,GQ279381.1,GQ279380.1,GQ279379.1,  JQ665912.1,JQ665911.1,JQ665910.1,FJ803217.1,FJ803214.1,FJ803208.1,FJ803206.1,FJ803205.1,FJ803202.1,FJ803216.1,FJ803215.1,FJ803213.1,FJ803212.1,FJ803211.1,FJ803210.1,FJ803207.1,FJ803201.1,AF288655.1,AF288653.1,AF288643.1,GQ274945.1,GQ274944.1,AB027522.1,  AF488707.1 |
| Amur virus | JX473004.1, EF121324.1, JX119010.1, KC136244.1, AB127997.1, AB127996.1, AB620028.1, AB071184.1, AB071183.1 |
| Gou virus | KC344252.1,KC344251.1,KC344250.1,JQ912802.1,JQ912801.1,  JQ912800.1,JQ912799.1,JQ912798.1,JQ912797.1,JQ912796.1,  JQ912795.1,JQ912794.1,JQ912793.1,JQ912792.1,JQ912791.1,  JQ912790.1,JQ912789.1,JQ912788.1,JQ912787.1,JQ912786.1,  JQ912785.1,JQ912784.1,JQ912783.1,JQ912782.1,JQ912781.1,  JQ912780.1,JQ912779.1,JQ912778.1,JQ912777.1,JQ912776.1,  JQ912775.1,JQ912774.1,JQ912773.1,JQ912772.1,JQ912771.1 |
| Andes virus | AF291702.1,AF324902.1,AY228237.1,AF004660.1,AF325966.1,  NC_003466.1 |
| Araucaria virus | AY740633.1,AY740630.1,AY740629.1,AY740628.1,AY740627.1,  AY740626.1,AY740625.1,AY740624.1, AY740623.1 |
| Bayou virus | L36929.1, GQ200820.1 |
| Laguna Negra virus | JX443704.1,JX443689.1,JX443688.1,JX443687.1,JX443686.1,  JX443684.1,JX443683.1, JX443682.1, JX443681.1 |
| Rio Mamore virus | JX443679.1,JX443678.1,JX443677.1,JX443676.1,JX443675.1,  JX443674.1,JX443673.1,JX443672.1,JX443671.1,JX443670.1,  JX443669.1 |
| Sin Nombre virus | JQ690278.1,JQ690277.1,JQ690276.1,L25784.1,L37904.1,NC_005216.1,  JQ690282.1,JQ690281.1 |
| Choclo virus | DQ285046 |
| Araraquara virus | EF571895 |
| Juquitiba virus | JX173798.2,KC422344.1,KC422345.1,KC422346.1,KC422347.1,  KC422348.1 |
|  |  |
| Virus Names | Accession numbers |
| Maripa virus | JQ611712.1,JN982967.1,KC876041.1 |
| Tula virus | AF164093.1,AF164094.1,AF289821.1,AF289820.1,AF289819.1,NC_005227.2,  AF063897.1,AF063892.1,AM945877.1,AF017659.1 |
| Puumala virus | JN657230.1,JN657229.1,JN657228.1,U14137.1,JN657232.1,JN657231.1,AF294652.1,NC_005224.1,KJ994776.1,AY526219.1,JQ319168.2,  JQ319163.2,JQ319162.2,Q319161.2,JQ319171.1,JQ319170.1,JQ319169.1,JQ319167.1,JQ319166.1,JQ319165.1,JQ319164.1,JN831950.1,JN831947.1,JN831943.1,AB433843.2,AB433845.2,GQ339487.1,GQ339486.1,GQ339485.1,GQ339484.1,GQ339483.1,GQ339482.1,GQ339481.1,GQ339480.1,GQ339479.1,GQ339478.1,GQ339477.1,GQ339476.1,GQ339475.1,  GQ339474.1,GQ339473.1,EF442087.1,JN696376.1,JN696375.1,  JN696374.1,JN696373.1,JN696372.1,JN696371.1,JN696358.1,  GU808825.1,GU808824.1,DQ016432.2,DQ016430.2,AF442613.1,  AF367071.1,AF367070.1,AF367069.1,AF367068.1,AF367067.1,  AF367066.1,AF367065.1,AF367064.1, AB010731.1,AB010730.1 |
| Cano Delgadito virus | DQ285566 |
| El Moro Canyon virus | U11427 |
| Montano virus | AB620100.1 |
| Muleshoe virus | U54575 |
| Prospect Hill virus | M34011.1 |
| Anjozorobe virus | KC490918.1,KC490917.1,KC490916.1,KC490915.1,KC490914.1 |
| Muju virus | JX028273.1,DQ138142.1,DQ138140.1,DQ138133.1,DQ138128.1 |
| Sangassou virus | JQ082303, JQ082300 |
| Thottapalayam virus | KJ420566.1,KJ420565.1,KJ420564.1,KJ420563.1,KJ420562.1,KJ420561.1, KJ420560.1,NC_010704.1,AY526097.1,JF784177.1,JF784176.1,JF784175.1,JF784174.1, JF784173.1,JF784172.1 |
| Asikkala virus | KC880343.1,KC880342.1,KC880341.1 |
| Cao Bang virus | EF543524 |
| Imjin virus | KJ420559.1,EF641805.1,EF641804.1 |
| Khabarovsk virus | U35255.1,KJ857346.1,KJ857345.1,KJ857344.1,KJ857343.1,KJ857342.1 |
| Longquan virus | JX465422.1,JX465421.1,JX465420.1,JX465419.1,JX465418.1,  JX465417.1,JX465416.1,JX465415.1,JX465414.1,JX465413.1 |
| Maporal virus | AY267347.1 |
| Rockport virus | HM015224.1,HM015223.1,HM015218.1 |
| Soochong virus | AY675352,AY675351,AY675350,AY675349 |
| Ussuri virus | AB677477.1,AB677476.1 |

Virus names with the accession numbers analyzed of the M segment

| Virus Names | Accession numbers |
| --- | --- |
| Hantaan virus | JQ083394.1,EF077656.1,U38177.1,U37729.1,AB030232.1,M14627.1,EU074672.1,AB027115.2,KT885048.1,AF366569.1,GQ120966.1,DQ371905.1,AF345636.2,EU363819.1,EU363818.1,EU363817.1,EU363816.1,  EU363815.1,EU363814.1,FJ753399.1,FJ753397.1,EF990929.1,  EF990928.1,EF990927.1,EF990926.1,EF990925.1,EF990924.1,  EF990923.1,EF990922.1,AF035831.1,D25532.1,D25529.1,D00377.1,  KJ857336.1,KJ857334.1,KC344264.1,KC344263.1,KC344262.1,  KC344261.1,KC344260.1,KC344259.1,KC344258.1,KC344257.1,  JQ912875.1,JQ912873.1,JQ912866.1,JQ912864.1,JQ912861.1,  JQ912859.1,JQ912856.1,JQ912854.1,JQ912852.1,JQ912851.1,  JQ912850.1,JQ912844.1,JQ912843.1,JQ912835.1,JQ912829.1,  JQ912828.1,JQ912825.1,JQ912824.1,JQ912818.1,JQ912814.1,  JQ912812.1,JQ912810.1,JQ912805.1,JQ912804.1,JQ912803.1,  JQ665882.1,JQ665881.1,AB620032.1,AF288645.1,AB127995.1,  EU074224.1,KC576787.1,KC576786.1 |
| Dobrava-Belgrade virus | JF920149.1,AY168578.1,AY168577.1,AY961616.1,NC_005234.1,  AJ410616.1,EU188453.1,EU188450.1,JQ026205.1,KT885042.1,GU904038.1,GU904037.1,GU904036.1,GU904034.1,GU904033.1,GQ205413.1,GQ205412.1,GQ205411.1,GQ205410.1,GQ205409.1,GU904035.1 |
| Seoul virus | KU204959.2,DQ133505.1,KP645197.1,JX853576.1,NC_005237.1,  KF387724.1,JX879768.1,KM948597.1,KM948593.1,AB618130.1,  S47716.1,KP859514.1,KP859513.1,D17594.1,D17593.1,D17592.1,  KM233661.1,KM233660.1,KM233659.1,KM233658.1,KM233657.1,  KM233656.1,KM233655.1,KM233654.1,KM233653.1,KM115585.1,  GU592931.1,GU592930.1,GU592929.1,GU592928.1,GU592927.1,  GU592926.1,GU592925.1,GU592924.1,GU592923.1,FJ811839.1,  KM115586.1,JQ665888.1,AF288654.1,AF288652.1,AF288650.1,  GQ274943.1,GQ274942.1,EF117248.1,DQ159911.1,AB027521.1 |
| Amur virus | JX473003.1,JX119009.1,EF371454.1,KC136243.1,AB127994.1,  AB127993.1,AB620029.1 |
| Gou virus | JQ912876.1,JQ912877.1,JQ912878.1,JQ912879.1,JQ912880.1,  JQ912881.1,JQ912882.1,JQ912885.1,JQ912886.1,JQ912887.1,  JQ912888.1,JQ912889.1,JQ912890.1,JQ912891.1,JQ912892.1,  JQ912893.1,JQ912894.1,JQ912895.1,JQ912896.1,JQ912897.1,JQ912898.1,JQ912899.1,JQ912900.1,JQ912901.1,JQ912902.1,JQ912903.1,  JQ912904.1,JQ912905.1,JQ912906.1,JQ912907.1,KC344267.1,  KC344268.1,KC344269.1 |
| Andes virus | AF324901.2,AF291703.2,AY228238.1,NC_003467.2 |
| Bayou virus | GQ244521,L36930.1 |
| Laguna Negra virus | JX443703 |
| Virus Names | Accession numbers |
| Sin Nombre virus | L37903.1,JQ690280.1,JQ690279.1,L25783.1,KT885045.1,KF537005.1,  KF537002.1,NC_005215.1 |
| Choclo virus | DQ285047 |
| New York virus | NYU36801,NYU36803,NYU36802 |
| Virus Names | Accession numbers |
| Maripa virus | JQ611714 |
| Tula virus | NC_005228 |
| Puumala virus | U14136.1,KJ994777.1,AY526218.1,NC_005223.1,KT885051.1,  JQ319175.1,JQ319174.1,JQ319173.1,JQ319172.1,JN831951.1,  JN831948.1,JN831944.1,AB433850.2,AB433852.2,AF442617.1,  AF442616.1,AF442615.1,AF442614.1,AF367061.1,L08754.1 |
| Cano Delgadito virus | DQ284451 |
| El Moro Canyon virus | EMU26828 |
| Montano virus | AB620101 |
| Anjozorobe virus | KC490921,KC490920,KC490919 |
| Muju virus | JX028272 |
| Sangassou virus | JQ082301 |
| Thottapalayam virus | KJ420548.1,KJ420546.1,KJ420545.1,KJ420544.1,KJ420543.1,  KJ420542.1,NC_010708.1,DQ825771.1,EU001329.1,JF784183.1,  JF784182.1,JF784181.1,JF784180.1,JF784179.1,JF784178.1 |
| Cao Bang virus | EF543526 |
| Imjin virus | EF641797,EF641798,EF641799 |
| Khabarovsk virus | KJ857340.1,KJ857339.1,KJ857338.1,KJ857332.1,KJ857331.1,  KJ857330.1,KJ857329.1 |
| Longquan virus | JX465396.1,JX465397.1,JX465398.1,JX465399.1,JX465400.1,  JX465401.1,JX465402.1 |
| Maporal virus | AY363179 |
| Soochong virus | AY675353.1,DQ056293.1,DQ056294.1,DQ056295.1 |
| Asikkala virus | KC880344,KC880345 |

Virus names with the accession numbers analyzed of the L segment

| Virus Names | Accession numbers |
| --- | --- |
| Andes virus | NC_003468,AF291704 |
| Amur virus | JX473002,KC136242,AB620030 |
| Bayou virus | GQ244526 |
| Laguna Negra virus | JX443696 |
| Sin Nombre virus | L37902,L37901,KT885044,KF537004,KF537001,NC_005217 |
| Cano Delgadito virus | GQ200821 |
| Montano virus | AB620102 |
| Dobrava virus | JQ026206,JF920148,NC_005235,AJ410617 |
| Seoul virus | KP645196,JX853574,KF387723,JX879770,NC_005238,KP900346,  KM948596,KM948595,KM948594,EF581094,EF190551 |
| Muju virus | JX028271 |
| Tula virus | NC_005226,AJ005637 |
| Puumala virus | AB574184,AB574183,EF405801,KJ994778,AY526217,KT885050,  JN831952,JN831949,JN831946,JN831945 |
| Sangassou virus | JQ082302 |
| Thottapalayam virus | KJ420576,KJ420575,KJ420574,KJ420573,KJ420570,KJ420569,  KJ420568,NC_010707,DQ825770,EU001330 |
| Black Creek Canal virus | GU997097 |
| Khabarovsk virus | KJ857322,KJ857321,KJ857315,KJ857314,KJ857313,KJ857312, KJ857311 |
| Prospect Hill virus | EF646763 |
| Rio Mamore virus | FJ809772 |
| Hantaan virus | KT885047,JQ083393,AF336826,NC_005222,DQ989237,DQ371906,KP896318,KP896317,KP896316,KP896315,KP896314,KP896313,KP896312,KP896311,KP896310,KP896309,KP896308,KP896307,  D25531,D25528,KJ857319,KJ857317,AB620033 |
| Maporal virus | EU788002 |
| Maripa virus | JQ611713 |
| Choclo virus | EF397003 |
| Soochong virus | AY675354,DQ056292 |
| Cao Bang virus | EF543525 |
| Imjin virus | EF641806,EF641807,KJ420567 |
| Rockport virus | HM015220,HM015221,HM015222 |

**Supplementary Table S2. Hantavirus genotypes classified based on their ability to cause human disease**

| **Virus Name** | **Number of sequences analysed** | **Old /New World hantaviruses** | **Human Disease** | **References** |
| --- | --- | --- | --- | --- |
| **Andes virus** | 12 | **New World Hantaviruses** | Both HCPS and HFRS | 1,7,19 |
| **Laguna Negra virus** | 11 | **New World Hantaviruses** | HCPS | 3,7 |
| **Araucaria virus** | 9 | **New World Hantaviruses** | HCPS | 7 |
| **Rio Mamore virus** | 13 | **New World Hantaviruses** | HCPS | 3,7 |
| **Bayou virus** | 5 | **New World Hantaviruses** | Both HCPS and HFRS | 8,11,13 |
| **Sin Nombre virus** | 22 | **New World Hantaviruses** | HCPS | 9,12 |
| **Choclo virus** | 3 | **New World Hantaviruses** | HCPS | 2,24 |
| **Araraquara virus** | 1 | **New World Hantaviruses** | HCPS | 3,4,5,7 |
| **Juquitiba virus** | 6 | **New World Hantaviruses** | HCPS | 3,5,6 |
| **Jabora virus** | 3 | **New World Hantaviruses** | HCPS | 3 |
| **Maripa virus** | 5 | **New World Hantaviruses** | HCPS | 7 |
| **Black Creek Canal virus** | 1 | **New World Hantaviruses** | Both HCPS and HFRS | 8,42 |
| **New York virus** | 3 | **New World Hantaviruses** | HCPS | 8 |
| **Puumala virus** | 94 | **Old World Hantaviruses** | Both HCPS and HFRS | 10,41 |
| **Tula virus** | 13 | **Old World Hantaviruses** | Both HCPS and HFRS | 18,20,41 |
| **Dobrava-Belgrade virus** | 66 | **Old World Hantaviruses** | HFRS | 14 |
| **Hantaan virus** | 248 | **Old World Hantaviruses** | HFRS | 15,16 |
| **Amur virus** | 19 | **Old World Hantaviruses** | HFRS | 25 |
| **Seoul virus** | 146 | **Old World Hantaviruses** | HFRS | 17 |
| **Gou virus** | 68 | **Old World Hantaviruses** | HFRS | 32 |
| **Thottapalayam virus** | 40 | **Old World Hantaviruses** | Not reported | 21,22 |
| **Prospect Hill virus** | 2 | **Old World Hantaviruses** | Not reported | 21 |
| **Sangassou virus** | 4 | **Old World Hantaviruses** | Not reported | 23 |
| **Anjozorobe virus** | 11 | **Old World Hantaviruses** | Not reported | 26 |
| **Muju virus** | 7 | **Old World Hantaviruses** | Not reported | 27 |
| **Cano Delgadito virus** | 3 | **Old World Hantaviruses** | Not reported | 30 |
| **Khabarovosk virus** | 20 | **Old World Hantaviruses** | Not reported | 25 |
| **Longquan virus** | 17 | **Old World Hantaviruses** | Not reported | 33 |
| **Soochong virus** | 10 | **Old World Hantaviruses** | Not reported | 34 |
| **Asikkala virus** | 5 | **Old World Hantaviruses** | Not reported | 35 |
| **Cao Bang virus** | 3 | **Old World Hantaviruses** | Not reported | 36 |
| **Montano virus** | 3 | **New World Hantaviruses** | Not reported | 31 |
| **Imjin virus** | 9 | **New World Hantaviruses** | Not reported | 37 |
| **Ussuri virus** | 2 | **New World Hantaviruses** | Not reported | 38 |
| **Rockport virus** | 6 | **New World Hantaviruses** | Not reported | 39 |
| **Maporal virus** | 3 | **New World Hantaviruses** | Not reported | 40 |
| **El Moro Canyon virus** | 2 | **New World Hantaviruses** | Not reported | 28 |
| **Muleshoe virus** | 1 | **New World Hantaviruses** | Not reported | 29 |

**References:**

1. Vial PA, Valdivieso F, Calvo MI et al; Hantavirus Study Group in Chile. A non-randomized multicentre trial of human immune plasma for treatment of hantavirus cardiopulmonary syndrome caused by Andes virus. *Antivir Ther* 2015; **20**:377-86.

2. Nelson R, Cañate R, Pascale JM et al. Confirmation of Choclo virus as the cause of hantavirus cardiopulmonary syndrome and high serum antibody prevalence in Panama. *J Med Virol* 2010; **82**:1586-93.

3. Guterres A, de Oliveira RC, Fernandes J, Schrago CG, de Lemos ER. Detection of different South American hantaviruses. *Virus Res* 2015; **210**:106-13.

4. Limongi JE, Oliveira RC, Guterres A et al. Hantavirus pulmonary syndrome and rodent reservoirs in the savanna-like biome of Brazil's southeastern region. *Epidemiol Infect* 2016; **144**:1107-16.

5. de Araujo J, Duré AI, Negrão R, Ometto T, Thomazelli LM, Durigon EL. Co-circulation in a single biome of the Juquitiba and Araraquara hantavirus detected in human sera in a sub-tropical region of Brazil. *J Med Virol* 2015; **87**:725-32.

6. Oliveira RC, Sant'ana MM, Guterres A et al. Hantavirus pulmonary syndrome in a highly endemic area of Brazil. *Epidemiol Infect* 2016; **144**:1096-106.

7. Figueiredo LT, Souza WM, Ferrés M, Enria DA. Hantaviruses and cardiopulmonary syndrome in South America. *Virus Res* 2014; **187**:43-54.

8. Knust B, Rollin PE. Twenty-year summary of surveillance for human hantavirus infections, United States. *Emerg Infect Dis* 2013; **19**:1934-7.

9. Rivers MN, Alexander JL, Rohde RE, Pierce JR Jr. Hantavirus pulmonary syndrome in Texas: 1993-2006. *South Med J* 2009; **102**:36-41.

10. Caramello P, Canta F, Bonino L et al. Puumala virus pulmonary syndrome in a Romanian immigrant. *J Travel Med* 2002; **9**:326-9.

11. Hjelle B, Goade D, Torrez-Martinez N et al. Hantavirus pulmonary syndrome, renal insufficiency, and myositis associated with infection by Bayou hantavirus. *Clin Infect Dis* 1996; **23**:495-500.

12. Khan AS, Khabbaz RF, Armstrong LR et al. Hantavirus pulmonary syndrome: the first 100 US cases. *J Infect Dis* 1996; **173**:1297-303.

13. Torrez-Martinez N, Bharadwaj M, Goade D et al. Bayou virus-associated hantavirus pulmonary syndrome in Eastern Texas: identification of the rice rat, Oryzomys palustris, as reservoir host. *Emerg Infect Dis* 1998; **4**:105-11.

14. Emmerich P, Müller N, Heinemann P et al. Human Dobrava-Belgrade hantavirus infection, Kosovo. *J Clin Virol* 2014; **61**:439-41.

15. Wang T, Liu J, Zhou Y et al. Prevalence of hemorrhagic fever with renal syndrome in Yiyuan County, China, 2005-2014. *BMC Infect Dis* 2016; **16**:69.

16. Yi J, Xu Z, Zhuang R et al. Hantaan virus RNA load in patients having hemorrhagic fever with renal syndrome: correlation with disease severity. *J Infect Dis* 2013; **207**:1457-61.

17. Macé G, Feyeux C, Mollard N et al. Severe Seoul hantavirus infection in a pregnant woman, France, October 2012. *Euro Surveill* 2013; **18**:20464.

18. Zelená H, Mrázek J, Kuhn T. Tula hantavirus infection in immunocompromised host, Czech Republic. *Emerg Infect Dis* 2013; **19**:1873-5.

19. Galeno H, Mora J, Villagra E et al. First human isolate of Hantavirus (Andes virus) in the Americas. *Emerg Infect Dis* 2002 ;**8**:657-61.

20. Reynes JM, Carli D, Boukezia N, Debruyne M, Herti S. Tula hantavirus infection in a hospitalised patient, France, June 2015. *Euro Surveill* 2015; 20(50).

21. Shim SH, Park MS, Moon S et al. Comparison of innate immune responses to pathogenic and putative non-pathogenic hantaviruses in vitro. *Virus Res* 2011; **160**:367-73.

22. Song JW, Baek LJ, Schmaljohn CS, Yanagihara R. Thottapalayam virus, a prototype shrewborne hantavirus. *Emerg Infect Dis* 2007; **13**:980-5.

23. Klempa B, Witkowski PT, Popugaeva E et al. Sangassou virus, the first hantavirus isolate from Africa, displays genetic and functional properties distinct from those of other murinae-associated hantaviruses. *J Virol* 2012; **86**:3819-27.

24. Atkinson B, Jameson LJ, Bovill BA et al. A non-fatal case of hantavirus cardiopulmonary syndrome imported into the UK (ex Panama), July 2014. *J Clin Virol* 2015; **67**:52-5.

25. Hörling J, Chizhikov V, Lundkvist Å et al. Khabarovsk virus: a phylogenetically and serologically distinct hantavirus isolated from Microtus fortis trapped in far-east Russia. *J Gen Virol* 1996; **77**:687-94.

26. Reynes JM, Razafindralambso NK, Lacoste V et al. Anjozorobe hantavirus, a new genetic variant of Thailand virus detected in rodents from Madagascar. *Vector Borne Zoonotic Dis* 2014; **14**:212-9.

27. Lee JG, Gu SH, Baek LJ et al. Muju virus, harbored by Myodes regulus in Korea, might represent a genetic variant of Puumala virus, the prototype arvicolid rodent-borne hantavirus. *Viruses* 2014; 6:1701-14.

28. Calisher CH, Root JJ, Mills JN et al. Epizootiology of Sin Nombre and El Moro Canyon hantaviruses, southeastern Colorado, 1995-2000. *J Wildl Dis* 2005; **41**:1-11.

29. Rawlings JA, Torrez-Martinez N, Neill SU et al. Cocirculation of multiple hantaviruses in Texas, with characterization of the small (S) genome of a previously undescribed virus of cotton rats (Sigmodon hispidus). *Am J Trop Med Hyg* 1996; **55**:672-9.

30. Milazzo ML, Duno G, Utrera A et al. Natural host relationships of hantaviruses native to western Venezuela. *Vector Borne Zoonotic Dis* 2010; **10**:605-11.

31. Saasa N, Sánchez-Hernández C, de Lourdes Romero-Almaraz M et al. Ecology of hantaviruses in Mexico: genetic identification of rodent host species and spillover infection. *Virus Res* 2012; **168**:88-96.

32. Wang W, Wang MR, Lin XD et al. Ongoing spillover of Hantaan and Gou hantaviruses from rodents is associated with hemorrhagic fever with renal syndrome (HFRS) in China. *PLoS Negl Trop Dis* 2013; **7**:e2484.

33. Guo WP, Lin XD, Wang W et al. Phylogeny and origins of hantaviruses harbored by bats, insectivores, and rodents. *PLoS Pathog* 2013; **9**:e1003159.

34. Baek LJ, Kariwa H, Lokugamage K et al. Soochong virus: an antigenically and genetically distinct hantavirus isolated from Apodemus peninsulae in Korea. *J Med Virol* 2006; **78**:290-7.

35. Radosa L, Schlegel M, Gebauer P et al. Detection of shrew-borne hantavirus in Eurasian pygmy shrew (Sorex minutus) in Central Europe. *Infect Genet Evol* 2013; **19**:403-10.

36. Gu SH, Arai S, Yu HT, Lim BK, Kang HJ, Yanagihara R. Genetic variants of Cao Bang hantavirus in the Chinese mole shrew (Anourosorex squamipes) and Taiwanese mole shrew (Anourosorex yamashinai). *Infect Genet Evol* 2016; **40**:113-8.

37. Lin XD, Zhou RH, Fan FN et al. Biodiversity and evolution of Imjin virus and Thottapalayam virus in Crocidurinae shrews in Zhejiang Province, China. *Virus Res* 2014; **189**:114-20.

39. Kang HJ, Bennett SN, Hope AG, Cook JA, Yanagihara R. Shared ancestry between a newfound mole-borne hantavirus and hantaviruses harbored by cricetid rodents. *J Virol* 2011; **85**:7496-503.

40. Fulhorst CF, Cajimat MN, Utrera A, Milazzo ML, Duno GM. Maporal virus, a hantavirus associated with the fulvous pygmy rice rat (Oligoryzomys fulvescens) in western Venezuela. *Virus Res* 2004; **104**:139-44.

41. Rasmuson J, Andersson C, Norrman E et al. Time to revise the paradigm of hantavirus syndromes? Hantavirus pulmonary syndrome caused by European hantavirus. *Eur J Clin Microbiol Infect Dis* 2011; **30**:685-90.

42. Klein SL, Calisher CH. Emergence and persistence of hantaviruses. *Curr Top Microbiol Immunol* 2007;315:217–52.

| **HCPS(n^1^)** | **Length** | **HFRS (n^1^)** | **Length** | **Both HFRS and HCPS (n^1^)** | **Length** | **No human disease reported (n^1^)** | **Length** |
| --- | --- | --- | --- | --- | --- | --- | --- |
| Laguna Negra virus (9) | 551.78 | Dobrava-Belgrade virus (41) | 318.05 | Puumala virus (64) | 477.56 | Thottapalayam virus(15) | 160.46 |
| Araucaria virus (9) | 574.56 | Hantaan virus (149) | 369.68 | Tula virus (10) | 503.8 | Prospect Hill virus (1) | 331 |
| Rio Mamore virus (11) | 618 | Amur virus (9) | 365.67 | Andes virus (6) | 545.66 | Sangassou virus(2) | 411.5 |
| Sin Nombre virus (8) | 712.62 | Seoul virus (89) | 416.12 | Bayou virus(2) | 628.5 | Anjozorobe virus(5) | 503.8 |
| Choclo virus (1) | 643 | Gou virus (35) | 442.23 |  |  | Muju virus(5) | 506.5 |
| Araraquara virus (1) | 529 |  |  |  |  | El Moro Canyon virus(1) | 567 |
| Jubiquito virus (6) | 571.17 |  |  |  |  | Muleshoe virus(1) | 660 |
| Jabora virus (3) | 469.33 |  |  |  |  | Cano Delgadito virus(1) | 660 |
| Maripa virus (3) | 602.33 |  |  |  |  | Montano virus(1) | 686 |
|  |  |  |  |  |  | Khabarovosk virus(6) | 501 |
|  |  |  |  |  |  | Longquan virus(10) | 236.6 |
|  |  |  |  |  |  | Soochong virus(4) | 369.5 |
|  |  |  |  |  |  | Asikkala virus(3) | 208.67 |
|  |  |  |  |  |  | Cao Bang virus(1) | 508 |
|  |  |  |  |  |  | Imjin virus(3) | 194.67 |
|  |  |  |  |  |  | Ussuri virus(2) | 452 |
|  |  |  |  |  |  | Maporal virus(1) | 530 |
|  |  |  |  |  |  | Rockport virus(3) | 511 |
| **Mean** | **597.06** |  | **383.67** |  | **489.43** |  | **346.75** |
| **SD** | **63.42** |  | **54.95** |  | **52.92** |  | **163.57** |

**Supplementary Table S3: Average length of 3’NCR of S-segment, M-segment and L-segment of each hantavirus genotype**

**Average length of 3’NCR of S-segment of each hantavirus genotype**

**^1^n=number of sequences**

**Average length of 3’NCR of M-segment of each hantavirus genotype**

| **HCPS (n^1^)** | **Length** | **HFRS (n^1^)** | **Length** | **Both HFRS and HCPS (n^1^)** | **Length** | **No human disease reported (n^1^)** | **Length** |
| --- | --- | --- | --- | --- | --- | --- | --- |
| Sin Nombre virus(8) | 168.25 | Seoul virus(46) | 201.82 | Puumala virus (20) | 179.25 | Anjozorobe virus(3) | 156.66 |
| Laguna Negra virus (1) | 217 | Amur virus(7) | 154.14 | Tula virus (1) | 213 | Muju virus(1) | 165 |
| Choclo virus(1) | 218 | Hantaan virus(76) | 164.48 | Bayou virus(2) | 195 | Montano virus(1) | 191 |
| Maripa virus(1) | 219 | Dobrava-Belgrade virus(21) | 189.71 | Andes virus(4) | 200 | Thottapalayam virus (15) | 192.2 |
| Rio Mamore virus (1) | 226 | Gou virus(33) | 203.03 |  |  | Sangassou virus(1) | 203 |
|  |  |  |  |  |  | Cano Delgadito virus(1) | 206 |
|  |  |  |  |  |  | El Moro Canyon virus(1) | 330 |
|  |  |  |  |  |  | Cao Bang virus(1) | 177 |
|  |  |  |  |  |  | Imjin virus(3) | 226 |
|  |  |  |  |  |  | Khabarovsk virus(7) | 222 |
|  |  |  |  |  |  | Longquan virus(7) | 197.28 |
|  |  |  |  |  |  | Maporal virus(1) | 218 |
|  |  |  |  |  |  | Soochong virus(4) | 167 |
|  |  |  |  |  |  | Asikkala virus(2) | 168 |
|  |  |  |  |  |  | New York virus(3) | 194 |
| **Mean** | **185.5** |  | **183.08** |  | **184.74** |  | **196.72** |
| **SD** | **73.15** |  | **22.41** |  | **37.14** |  | **37.74** |

**^1^n=number of sequences**

**Average length of 3’NCR of L-segment of each hantavirus genotype**

| **HCPS (n^1^)** | **Length** | **HFRS (n^1^)** | **Length** | **HFRS/HCPS (n^1^)** | **Length** | **No human disease reported (n^1^)** | **Length** |
| --- | --- | --- | --- | --- | --- | --- | --- |
| Sin Nombre virus(6) | 61 | Dobrava-Belgrade virus (4) | 39.5 | Puumala virus (10) | 43 | Anjozorobe virus(3) | 16 |
| Laguna Negra virus (1) | 65 | Hantaan virus (23) | 39.13 | Tula virus(2) | 43 | Sangassou virus (1) | 38 |
| Choclo virus(1) | 65 | Amur virus(3) | 22.33 | Black Creek Canal virus(1) | 61 | Muju virus(1) | 43 |
| Maripa virus(1) | 66 | Seoul virus (11) | 37.27 | Andes virus(2) | 65 | Montano virus(1) | 54 |
| Rio Mamore virus (1) | 66 |  |  | Bayou virus( 1) | 62 | Prospect Hill virus(1) | 55 |
|  |  |  |  |  |  | Thottapalayam virus (10) | 65 |
|  |  |  |  |  |  | Cano Delgadito virus(1) | 73 |
|  |  |  |  |  |  | Maporal virus(1) | 66 |
|  |  |  |  |  |  | Soochong virus(2) | 40 |
|  |  |  |  |  |  | Cao Bang virus(1) | 39 |
|  |  |  |  |  |  | Imjin virus(3) | 79.66 |
|  |  |  |  |  |  | Rockport virus(3) | 53 |
|  |  |  |  |  |  | Khabarovsk virus(7) | 75.14 |
| **Mean** | **62.8** |  | **37.43** |  | **43** |  | **59.14** |
| **SD** | **5.26** |  | **6.53** |  | **9.1** |  | **18.25** |

**^1^n=number of sequences**

**Supplementary Table S4. Average length of the 5' NCR**

| **5'NCR segment** | **Length of 5'NCR in HCPS-causing genotypes: Mean + SD (n=number of sequences)** | **Length of 5'NCR in HFRS-causing genotypes: Mean + SD (n=number of sequences)** | **P Value** |
| --- | --- | --- | --- |
| **S-segment**  **M-segment**  **L-segment** | 36.43 ± 8.70 (51)  52 ± 20.37 (12)  30.44 ± 8.76 (9) | 35.46 ± 8.23 (319)  42.34 ± 5.47 (181)  35.07 ± 5.38 S(41) | 0.43  <0.001  0.04 |
