# Supplementary figures and images for: Identification of tell-tale patterns in the 3′ non-coding region of hantaviruses that distinguish HCPS-causing hantaviruses from HFRS-causing hantaviruses
Source: Emerg Microbes Infect. 2018 Mar 21;7:32. doi: 10.1038/s41426-018-0027-z (PMC5861111; doi:10.1038/s41426-018-0027-z)

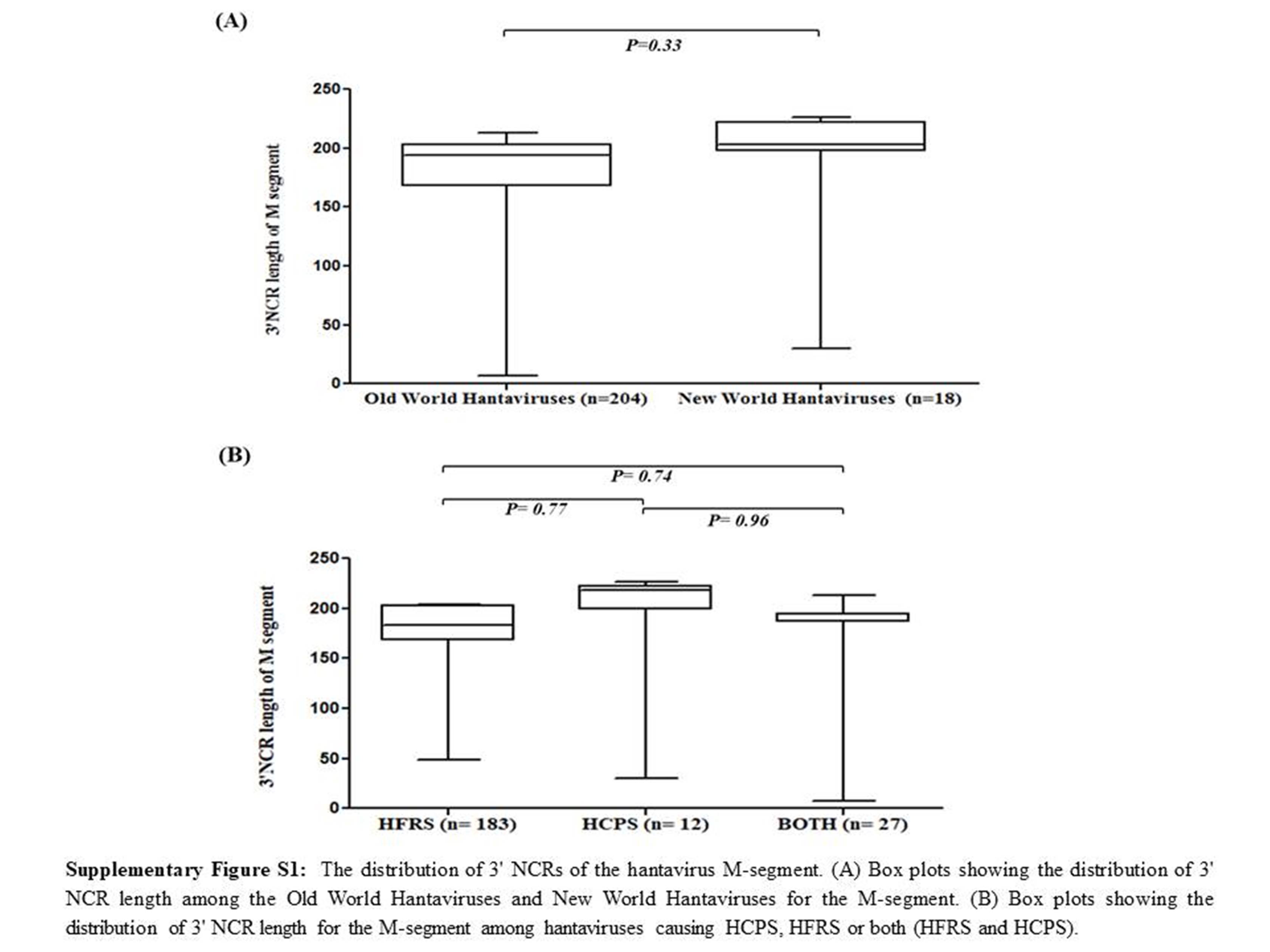

Supplement: Supplementary file 2 — Supplementary Figure S1 [file 41426_2018_27_MOESM2_ESM.jpg]

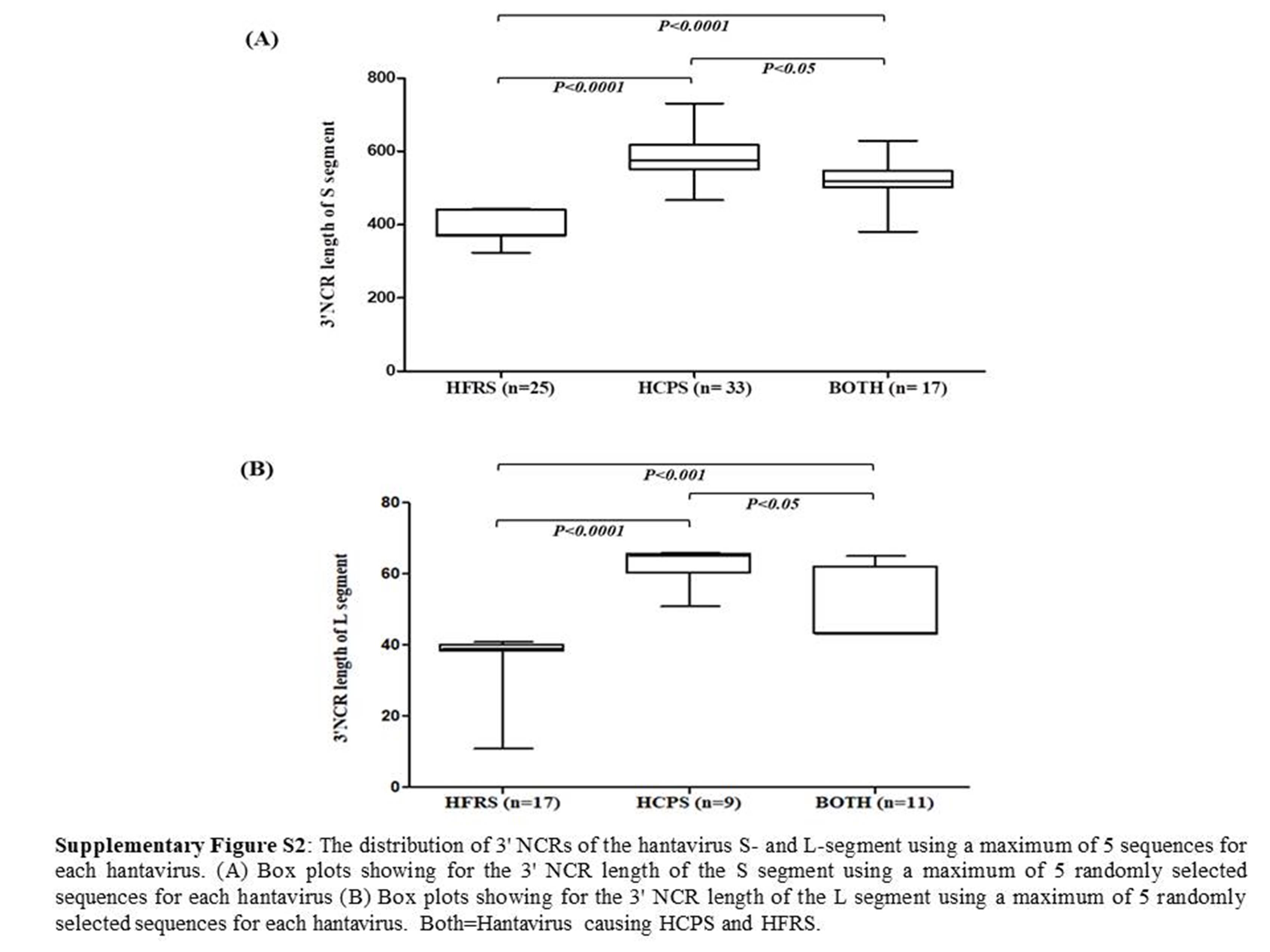

Supplement: Supplementary file 3 — Supplementary Figure S2 [file 41426_2018_27_MOESM3_ESM.jpg]

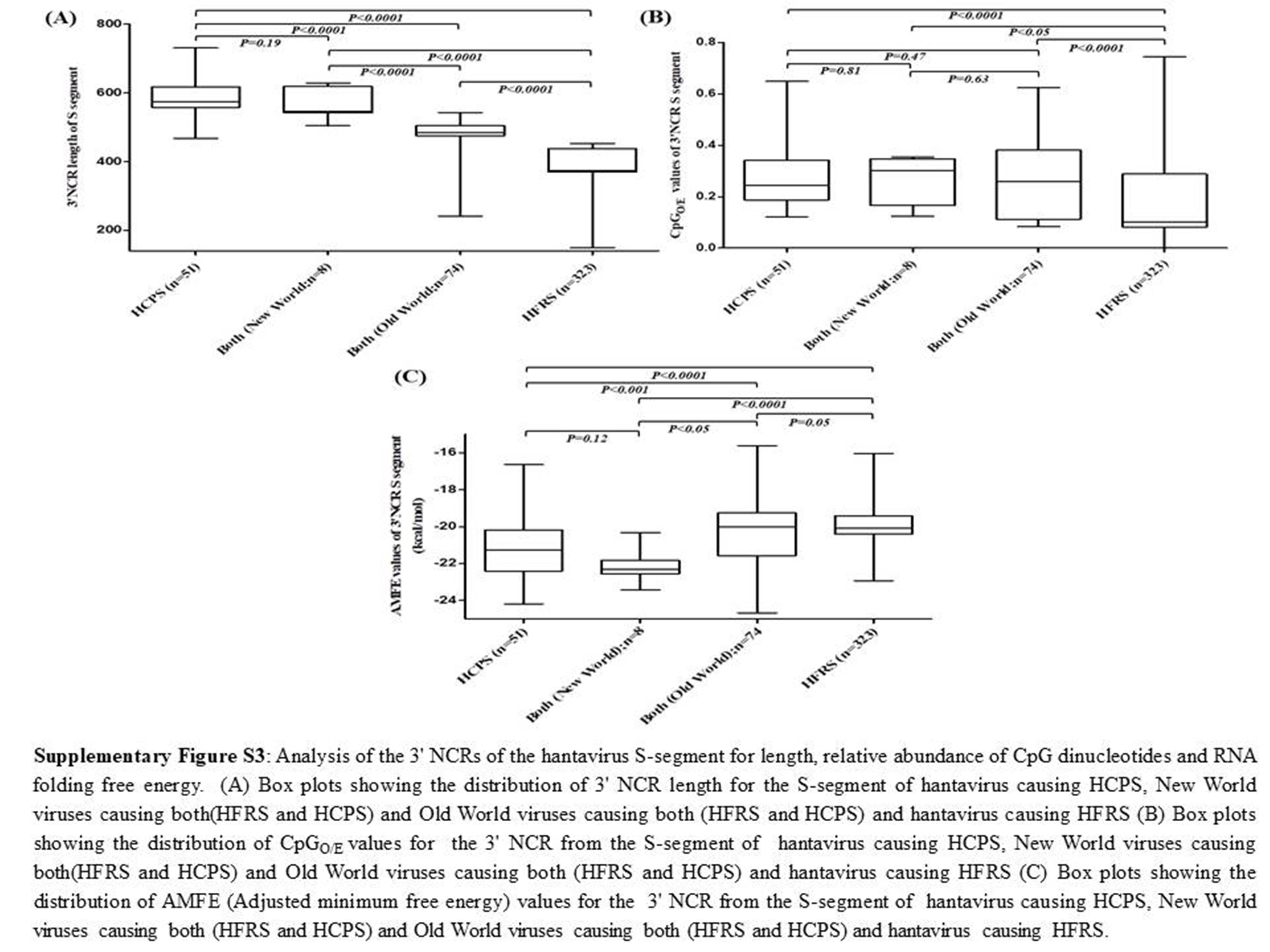

Supplement: Supplementary file 4 — Supplementary Figure S3 [file 41426_2018_27_MOESM4_ESM.jpg]
